# First Spaceborne SAR-GMTI Experimental Results for the Chinese Gaofen-3 Dual-Channel SAR Sensor

**DOI:** 10.3390/s17112683

**Published:** 2017-11-21

**Authors:** Chenghao Wang, Guisheng Liao, Qingjun Zhang

**Affiliations:** 1 National Key Laboratory of Radar Signal Processing, Xidian University, Shaanxi 710071, China; wangchenghao1991@126.com (C.W.); gsliao@xidian.edu.cn (G.L.); 2 Beijing Institute of Spacecraft System Engineering, China Academy of Space Technology, Beijing 100094, China

**Keywords:** Gaofen-3, SAR-GMTI, azimuth ambiguity, clutter suppression, vector velocity estimation

## Abstract

In spaceborne synthetic aperture radar (SAR) sensors, it is a challenging task to detect ground slow-moving targets against strong clutter background with limited spatial channels and restricted pulse repetition frequency (PRF). In this paper, we evaluate the image-based dual-channel SAR-ground moving target indication (SAR-GMTI) workflow for the Gaofen-3 SAR sensor and analyze the impact of strong azimuth ambiguities on GMTI when the displaced phase center antenna (DPCA) condition is not fully satisfied, which has not been demonstrated yet. An effective sliding window design technique based on system parameters analysis is proposed to deal with azimuth ambiguities and reduce false alarm. In the SAR-GMTI experiments, co-registration, clutter suppression, constant false alarm rate (CFAR) detector, vector velocity estimation and moving target relocation are analyzed and discussed thoroughly. With the real measured data of the Gaofen-3 dual-channel SAR sensor, the GMTI capability of this sensor is demonstrated and the effectiveness of the proposed method is verified.

## 1. Introduction

Spaceborne synthetic aperture radar (SAR) sensors are capable of observing the earth surface continuously regardless of weather and daylight, which motivates the rapid development of spacecraft and SAR sensor technologies along with their wide applications in civil and military uses [1,2,3,4]. Moreover, the advanced multiple-channel SAR (MC-SAR) sensor technology leads the trend in spaceborne SAR roadmap. The along-track orientated MC-SAR sensor provides additional spatial degrees-of-freedom (DOFs) in azimuth, which makes it possible to implement digital beamforming (DBF) technique on receive [5]. Thus, the MC-SAR sensor can either be used to identify ground/maritime moving targets [3,4,5,6] or to reconstruct high-resolution and wide-swath (HRWS) SAR images [7].

It is a challenging task to detect ground slow-moving targets buried in strong ground clutter, while the MC-SAR-ground moving target indication (GMTI) technique deals with this problem in several aspects. On the one hand, SAR imaging improves the signal-to-noise ratio (SNR) and signal-to-clutter-plus-noise ratio (SCNR) via long-time integration and two-dimensional high resolution, respectively, where the latter isolates and narrows the clutter patch that competes with the moving targets. On the other hand, MC-SAR sensors provide multiple observations of the same scene at different time, which brings in clutter cancellation ability with dual-channel SAR sensors and additional accurate target radial velocity estimation with tri(or more)-channel SAR sensors [4,8]. However, it would be increasingly expensive and technically challenging to design and produce spaceborne SAR sensors with many spatial channels, so that a dual-channel structure is generally applied in state-of-art satellite SAR sensors, such as Radarsat-2, TerraSAR-X and Tandem-X. Nevertheless, it is still possible to achieve both clutter cancellation and target radial velocity estimation tasks with only two-channels, in condition that SNR and SCNR are not lower than some threshold depending on system configurations [4]. Luckily, this condition is not very hard to reach in spaceborne SAR for moving targets owing to the SNR and SCNR advantages mention above. Through the performance evaluation with the data in this paper, it is shown that the across-track ground velocity estimation accuracy could be better than 1 m/s with a high probability for targets with input SCNR higher than around 10 dB for Gaofen-3.

Clutter suppression is the core of the SAR-GMTI technique, which is done optionally in data domain or image domain. Data domain methods perform clutter suppression directly after range compression, such as space-time adaptive processing (STAP) [9,10,11] and imaging STAP (ISTAP) [12,13], which can be used for arbitrary number of channels. Post-Doppler STAP [14] is commonly adopted for MC-SAR-GMTI, considering that sufficient pulses are available while spatial channels are limited, and also that this technique can provide near-optimal STAP performance with reduced dimension and computational burden. However, only part of the aperture $\sqrt{2\lambda R}$ would be coherently integrated if detection procedure were directly done after Post-Doppler STAP. Moreover, if the STAP filtered data are imaged using SAR algorithms and then fed into a constant false alarm rate (CFAR) detector, great SNR gain would be obtained for moving targets but optimal SNR and SCNR gain are still not achieved due to smearing effect caused by target motion. To solve this problem, ISTAP inserts azimuth matched filters between STAP and CFAR detection procedures to image (focus) the targets so as to further improve the SCNR before detection; however, computational burden is increased a lot. Image domain methods mainly involves along-track interferometry (ATI) [15,16], displaced phase center antenna (DPCA) [17], extended DPCA (EDPCA) [12,18], signal subspace projection (SSP) [19,20], and joint pixel vector method (JPVM) [21,22,23]. ATI and DPCA approaches are originally developed for dual-channel SAR sensors and calls for precise co-registration and imbalance correction. Based on DPCA approach, the EDPCA, SSP and JPVM are extendable to more channels, where EDPCA aims at focusing targets and achieving optimal output SCNR, while SSP and JPVM improve robustness to co-registration error and channel imbalance. However, their adaptive processing needs training samples, which may result in degradation in heterogeneous environment [24]. Actually, for images that are well co-registered and balanced (achieved by sensor calibration or signal processing algorithms [25,26]), DPCA is a deterministic approach and free of training so as to cancel strong stationaries in heterogeneous environment as well. Note that the digital balancing (DB) algorithm developed in [25] and updated in [3,26,27] performs channel balancing and co-registration (along- and across-track) at the same time, for raw as well as focused data.

In spaceborne SAR, not only the spatial channels are limited, the pulse repetition frequency (PRF) is also constrained to many factors. Due to the fast speed of the platform, PRF is usually not allowed to be very high in compromise with the swath width. This leads to increased azimuth ambiguity-to-signal ratio (AASR) compared to that in airborne case where PRF is relative marginal to cover the first high sidelobe. Though AASR can be controlled via PRF design to avoid image quality degradation, the azimuth ambiguities would still appear in the clutter canceled image for dual-channel SAR sensors when the DPCA condition is not fully satisfied [28], thus influencing the moving target detection. Note that the DPCA condition is not always satisfied in compromise with AASR and azimuth resolution of the SAR imagery, so that it is important to study the impact of azimuth ambiguities on GMTI when the DPCA condition is not met and to find out techniques that can mitigate them.

In this paper, we investigate the dual-channel SAR-GMTI methods for spaceborne SAR sensors and analyze the GMTI experiment of the Gaofen-3 satellite. Several image-based clutter suppression methods are compared using real measured data. Additionally, azimuth ambiguous clutter is analyzed and a parameter chosen technique for CFAR detector is proposed based on system parameters and image feature of the strong azimuth ambiguous clutter, which is shown to be capable of mitigating false alarm of the azimuth ambiguities to improve vehicle detection performance effectively. Section 2 describes the geometry and signal model for Gaofen-3 SAR sensor with dual-channel GMTI experimental mode. Section 3 presents several image-based SAR-GMTI methods and overall workflow. Section 4 gives the GMTI experimental results and some discussions on them. Finally, several conclusions are drawn in Section 5.

## 2. Geometry and Signal Model

### 2.1. Gaofen-3 SAR Sensor and Data Acquisition

Gaofen-3 SAR sensor is the first Chinese fully polarimetric C-band SAR satellite [29], which was launched in 10 August 2016. The Gaofen-3 SAR sensor is designed with full polarization capability and dual-receive channel architecture. The resolution of Gaofen-3 SAR sensor ranges from 1 m to 500 m, while the corresponding swath ranges from 10 km up to 650 km. Thus far, 12 imaging modes [29,30,31,32] have been designed and successfully implemented on this sensor.

This sensor is equipped with a wave-guide slot phased array with length 15 m and height 1.5 m [29,32], which can be divided into two halves in azimuth on reception. Based on this architecture, a dual receive channel (DRC) mode based on ultra-fine strip (UFS) mode has been implemented on Gaofen-3 for HRWS imaging of static scene and moving targets purposes [33]. This design employs a PRF lower than the Doppler bandwidth, so that the two spatial channels are used to resolve the azimuth ambiguity before unambiguous imaging of the scene or moving targets such as vessels with sufficient SCNR. However, there are no more spatial DOFs available to further perform clutter cancellation, which would limit its GMTI capability greatly, especially for weak moving targets such as vehicles and small boats. For this sake, a DRC GMTI experimental mode is designed based on fine strip I (FSI) mode with nominal resolution of 5 m, where the PRF is designed larger than the Doppler bandwidth to ensure unambiguous imaging of the scene, so that the spatial DOFs can be used for GMTI purpose.

**Figure 1:**
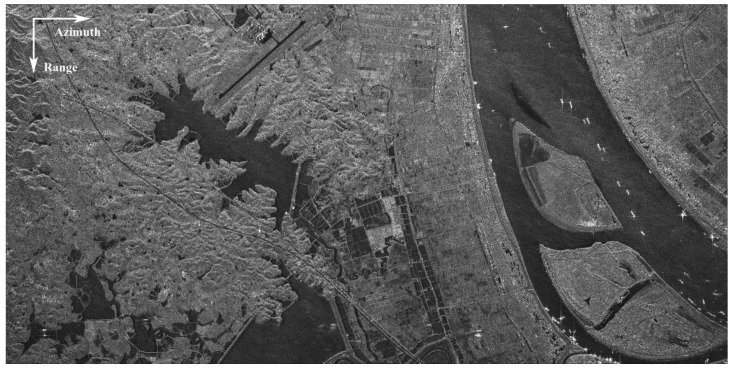
Imaging scene for synthetic aperture radar-ground moving target indication (SAR-GMTI) experiment.

For SAR-GMTI evaluation purposes, a scene has been selected over the Chinese Yangtze River as shown in Figure 1. Note that left part of the scene that contains a highway is the area of interest (AOI) for SAR-GMTI purpose. The main acquisition parameters are listed in Table 1, where the nominal Doppler bandwidth is given here in accordance with the standard FSI mode; however, the real one would be a little higher for DRC mode, due to a wider receive beam pattern with only half of the full array. It can be deduced from Table 1 that the unambiguous across-track ground velocity is around 48.8 m/s. During acquisition, the Doppler center of the clutter is locked to zero Hz by controlling the attitude of the satellite, i.e., the yaw angle.

### 2.2. DRC GMTI Experimental Mode Geometry

**Figure 2:**
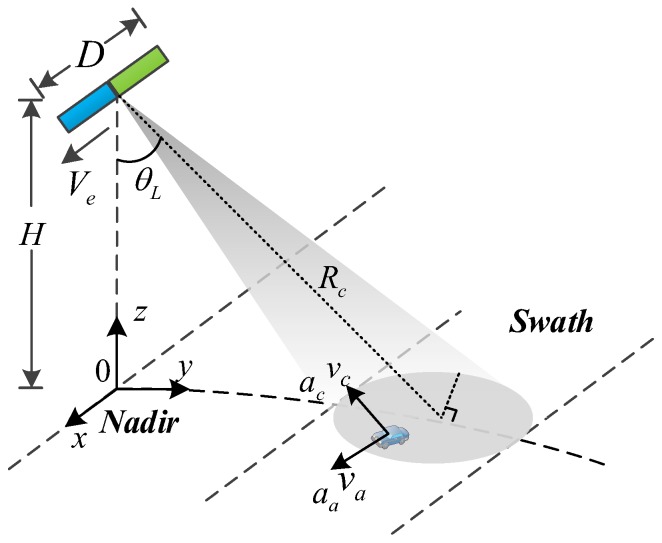
Geometry of the Gaofen-3 SAR sensor with dual receive channel mode.

In DRC mode, the Gaofe-3 SAR sensor transmits pulses with the full aperture length D and receives the echo with two sub-apertures simultaneously, where each sub-aperture is half of the full antenna and the two halves are placed along track. The geometry of the satellite is briefly shown in Figure 2, where the satellite flies along *x*-axis at the altitude H with the effective velocity $V_{e}\approx \sqrt{V_{s}V_{g}}$, where $V_{s}$ and $V_{g}$ are the satellite and ground velocities, respectively. The antenna beam is steered $\theta_{L}$ off the nadir, so that the observed scene is illuminated with the center slant range being $R_{c}$. The ground moving targets, such as vehicles and dismounts, are assumed to move on the ground where $v_{a}$ and $v_{c}$ are the along-track and across-track velocities in the slant plane, respectively; and $a_{a}$ and $a_{c}$ are the along-track and across-track accelerations, respectively. Note that $v_{c}$ is positive when a target is approaching the radar.

According to the equivalent phase center (EPC) principle, this DRC architecture is equivalent to the self-transmit self-received process of two EPCs, which are approximately located at the middle points between the transmit/receive pairs. Consequently, this results in a separation, namely baseline, of around $B_{x}\approx D/4$ between the two EPCs for DRC mode. Thus, it takes a time delay of τ for the aft-EPC traveling over the baseline to the same spatial position as the fore-EPC, which can be expressed as

(1) $$\tau =\frac{B_{x}}{V_{s}}=\frac{B_{xe}}{V_{e}}$$

where $B_{xe}=B_{x}\frac{V_{e}}{V_{s}}$ is the effective baseline. Let $\left(R_{0},X_{0}\right)$ be the reference position of a ground moving target, where $R_{0}$ is the closest slant range and $X_{0}$ denotes its azimuth position at slow time $t_{a}=0$. Thus, the positions of the moving target and radar can be denoted as $\left(R_{0}-v_{c}t_{a}-\frac{1}{2}a_{c}t_{a}^{2},X_{0}+v_{a}t_{a}+\frac{1}{2}a_{a}t_{a}^{2}\right)$ and $\left(0,V_{e}t_{a}\right)$, respectively. In general, we take the fore-channel as the reference with position $\left(0,V_{e}t_{a}\right)$, so that the aft-channel position is defined as $\left(0,V_{e}t_{a}-B_{xe}\right)$. Consequently, the slant range equations can be obtained as

(2) $$R_{1}\left(t_{a}\right)=\sqrt{{\left(R_{0}-v_{c}t_{a}-\frac{1}{2}a_{c}t_{a}^{2}\right)}^{2}+{\left(V_{e}t_{a}-X_{0}-v_{a}t_{a}-\frac{1}{2}a_{a}t_{a}^{2}\right)}^{2}}$$

for fore-channel and

(3) $$R_{2}\left(t_{a}\right)=\sqrt{{\left(R_{0}-v_{c}t_{a}-\frac{1}{2}a_{c}t_{a}^{2}\right)}^{2}+{\left(V_{e}t_{a}-B_{xe}-X_{0}-v_{a}t_{a}-\frac{1}{2}a_{a}t_{a}^{2}\right)}^{2}}$$

for aft-channel.

### 2.3. Signal Model

The range-compressed echo signal of the ith channel is shown as

(4) $$S_{i}\left(t_{r},t_{a}\right)=\sigma G_{r}\mathrm{sinc}\left(B_{w}\left(t_{r}-R_{i}\left(t_{a}\right)\right)\right)\omega \left(t_{a}-\frac{X_{0}}{V_{e}}\right)\exp\left(-j\frac{4\pi R_{i}\left(t_{a}\right)}{\lambda }\right)$$

where σ is the complex amplitude of the target echo; $G_{r}$ is the gain of the matched filter; $B_{w}$ is the system bandwidth; $\omega \left(t_{a}\right)$ denotes the azimuth envelope, which depends on the antenna beam pattern; and λ is the wavelength. Two images can be formed after applying SAR imaging processing to Equation (4). Note that the two images are observed with an interval τ as shown in Equation (1). With the compensation of τ and channel-imbalance, the two images are co-registered and imbalance-corrected, which can be denoted as $s_{1}\left(m,n\right)$ for fore-channel and $s_{2}\left(m,n\right)$ for aft-channel, where m and n are the pixel indexes for range and azimuth, respectively.

Given the co-registered $s_{1}\left(m,n\right)$ and $s_{2}\left(m,n\right)$, each pair of pixels are essentially two interval sampling of the corresponding ground patch. Thus, the interferometric phase between two channels is derived as

(5) $$\begin{array}{cc} \Delta \phi & =\phi_{2}-\phi_{1}\\ & \approx {\frac{\partial \phi_{1}}{\partial t_{a}}|}_{t_{a}=t_{0}}\tau \\ & =\frac{4\pi B_{xe}}{\lambda V_{e}}v_{c} \end{array}$$

where $\phi_{1}$ and $\phi_{2}$ are the phase of $s_{1}\left(m,n\right)$ and $s_{2}\left(m,n\right)$, respectively, which are related to the last exponential term of the SAR signal phase history Equation (5); and $t_{0}=\frac{X_{0}}{V_{e}-v_{a}}$ is the instant when the radar is closest to the target, and also the center of the synthetic aperture approximately. It could be observed from Equation (5) that Δϕ is proportional to the across-track velocity $v_{c}$. Specifically, it has $v_{c}=0$ for stationary targets, which yields $\Delta \phi_{c}=0$ for main clutter. Note that Equation (5) is an approximation without considering the impact of across-track acceleration or along-track velocity, the impact of which has been investigated in [34].

In a single pixel, it may contain several components, including moving target, stationary clutter, and noise. Owing to the discrete sampling of the SAR signal (Equation (4)) by PRF in slow-time domain, the azimuth ambiguous clutter component should be considered when the PRF is limited, especially in spaceborne SAR. The interferometric phase of the azimuth ambiguities is derived as

(6) $$\Delta \phi_{c}^{l}=\frac{4\pi B_{xe}}{\lambda V_{e}}\frac{lf_{prf}\lambda }{2}=l\frac{2\pi B_{xe}f_{prf}}{V_{e}}$$

where *l* denotes the ambiguity number and $f_{prf}$ is the PRF. Specifically, l=0 corresponds the unambiguous main clutter. Allowing for the components mentioned above, we obtain the signal model in image domain as

(7) s=t+c+n

where $s={\left[\begin{array}{cc} s_{1}\left(m,n\right) & s_{2}\left(m,n\right) \end{array}\right]}^{T}$, ${\left(\cdot \right)}^{T}$ denotes the transpose operator. In Equation (7), the moving target component can be written as $t=\sigma_{t}{\left[\begin{array}{cc} 1 & e^{j\Delta \phi_{t}} \end{array}\right]}^{T}$, where $\sigma_{t}$ and $\Delta \phi_{t}$ are the complex amplitude and the interferometric phase of the target, respectively. In addition, the main clutter and the ambiguities are merged as

(8) $$c=\sum_{l,k}^{N}c_{k}^{l}$$

where *N* is the total number of range and azimuth ambiguity patches considered, and $c_{k}^{l}$ is the clutter vector for the *l*th azimuth and *k*th range ambiguity, which is assumed to have a stationary zero mean complex Gaussian distribution, i.e., $c_{k}^{l}\in \mathbb{C}∼N(0,R_{c,k}^{l})$ as extensively considered. Note that this assumption does not hold true for highly heterogeneous scene [16]. The covariance matrix of the *l,k*th ambiguous clutter is $R_{c,k}^{l}=\left[\begin{array}{cc} \sigma_{c1,l,k}^{2} & \rho_{c1,2}\sigma_{c1-l,k}\sigma_{c2-l,k}e^{j\Delta \phi_{c}^{l}}\\ \rho_{c1,2}\sigma_{c1-l,k}\sigma_{c2-l,k}e^{-j\Delta \phi_{c}^{l}} & \sigma_{c2-l,k}^{2} \end{array}\right]$ [4], where $\sigma_{ci-l,k}^{2}$ denotes the clutter power of the *l,k*th ambiguous clutter received by the *i*th channel, and $\rho_{c1,2}$ is the correlation coefficient between channel 1 and 2 which takes account temporal decorrelation induced by internal clutter motion and is assumed to be independent of ambiguous number for simplicity. In addition, the noise component is $n={\left[\begin{array}{cc} n_{1} & n_{2} \end{array}\right]}^{T}$, where $n_{1}$ and $n_{2}$ are assumed as independent identical distributed (IID) white Gaussian noise, so as to have a covariance matrix of $R_{n}=\sigma_{n}^{2}I$ with $\sigma_{n}^{2}$ being the noise power.

For spaceborne SAR sensor, ambiguities on range and azimuth are major concerns that influence the final performance non-negligibly, for both SAR imaging as well as GMTI mission. The clutter model presented above can be used to analysis the theoretical GMTI performance by means of covariance matrix modeling. To model the covariance matrix, total N range and azimuth ambiguous clutter patches should be considered in addition to the unambiguous main clutter patch. Assume that different patches are uncorrelated; the covariance matrix of clutter plus noise can be expressed as

(9) $$R_{c}=R_{c}{}^{0}+\sum_{l,k}^{N}R_{c,k}^{l}+R_{n},\;\;\left(l,k\right)\neq \left(0,0\right)$$

Note that $\sigma_{ci-l,k}^{2}$ can be expressed with respect to the power of main clutter patch $\sigma_{ci}^{2}$ using a SAR related metric, i.e., the combined-range-azimuth-ambiguity-to-signal ratio (CRAASR) as

(10) $$\sigma_{ci-l,k}^{2}=\sigma_{ci}^{2}\times {\mathrm{CRAASR}}_{l,k}$$

where ${\mathrm{CRAASR}}_{l,k}$ has been defined in [4]. The theoretical GMTI performance can be analyzed concerning the eigenvalue distribution of $R_{c}$, as well as the SCNR/probability of detection (*P_d_*) metrics under specific configuration and processing technique (such as DPCA, SSP, EDPCA, etc.) with respect to ground range velocity, target RCS, incidence angle, etc. The eigenvalue distribution of $R_{c}$ can be compared against the estimated one to validate the theoretical clutter model. While the SCNR/*P_d_* metrics are also closely related to the theoretical covariance matrix $R_{c}$, which is a statistical based metric of the clutter characteristics and thus exploited by various adaptive techniques.

## 3. Image Domain SAR-GMTI Method

**Figure 3:**
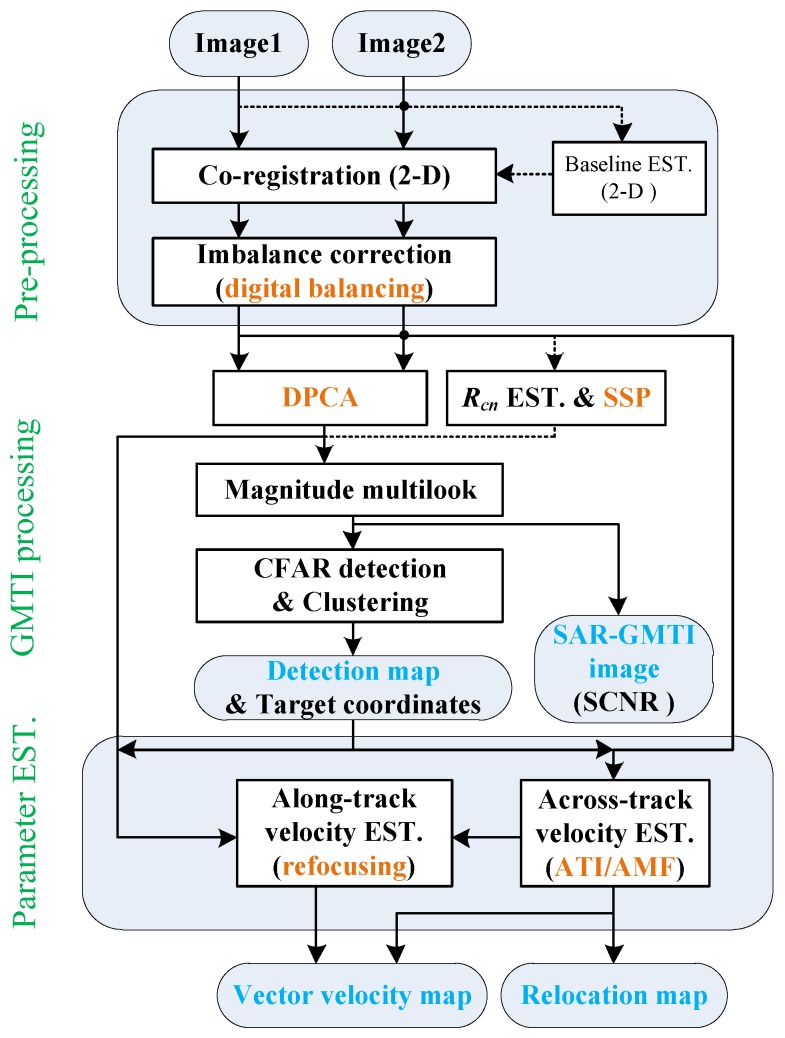
Workflow of the image-based SAR-GMTI processing.

The overall processing workflow is shown in Figure 3. Image 1 and Image 2 are the single-look complex (SLC) SAR images generated by aft-channel and fore-channel, respectively. The images are generated with neither azimuth nor range windowing to avoid attenuation of the moving target, especially the one with high radial velocity. Pre-processing stage includes co-registration and imbalance correction of the images. Co-registration removes the time delays of both along- and across-track baselines by optional baseline estimation in range frequency and Doppler domain. Imbalance correction equalizes the channel response to ensure a proper clutter cancellation, which is done using digital balancing technique as described in the Introduction. GMTI processing mainly involves clutter cancellation and target detection, where clutter cancellation is done alternatively by deterministic approach like DPCA or an adaptive way like SSP, which requires estimation of the covariance matrix in image domain. Note that the covariance matrix should be estimated from a region with adequate CNR and low number of moving targets to achieve proper clutter cancellation and avoid target suppression. Multilook is performed before CFAR detection to reduce the variance of the clutter statistic. Note that the multilook cell size should be no more than target dimension to avoid reduced SCNR of the target [16]. Then, potential targets are detected using CFAR detector. Across-track velocity of the target is estimated from ATI phase or via adaptive matched filter (AMF) using the images before clutter cancellation. Along-track velocity can be estimated by refocusing the target chip extracted from the clutter canceled image with the estimated across-track velocity as an input parameter. Finally, target relocation map and vector velocity map are obtained using the estimated parameters.

### 3.1. Baseline Estimation and Images Co-Registration

Generally, the co-registration accuracy of two images is crucial to image based SAR-GMTI methods such as DPCA and ATI, which usually requires co-registration accuracy much less than one pixel to obtain satisfactory clutter suppression ratio or to construct interferometric phase, which is especially true for high-resolution SAR sensors. However, the nominal baseline is generally not identical with the effective baseline, which is caused by various factors such as antenna position error during manufacturing, bias between array EPC and physical center, existing of across-track baseline component, etc. Note that the last factor is induced by array crab angle in airborne case. For Gaofen-3 satellite, a crab angle of the satellite is preset to counter the Doppler center induced by earth rotation. Thus, the effective baseline will be slightly shorter than the nominal one. Consequently, high precision co-registration and effective baseline estimation is necessary to improve the overall GMTI performance of the sensor.

The obtained two SAR images can be co-registered either by image correlation or by interferometric phase deramp in 2-D frequency domain [25,26]. The former requires image interpolation to achieve a co-registration error less than one pixel, while the latter method, also called 2-D adaptive calibration [25], is performed in the 2-D frequency domain to achieve both along- and across-track baseline delay compensation. The 2-D adaptive calibration proposed by Gierull in [25] can perform channel balancing and co-registration simultaneously. However, this method requires the data source to have sufficient CNR and avoid strong and/or large number of moving targets, which may degrade the estimation of the calibration weight. To deal with the degradation caused by moving targets, a revised version was also presented in [26] by taking only the smoothest region of the sorted interferometric phase for weight estimation, based on the observation that the moving targets are not likely to be distributed in this region. Thus, the phase imbalances can be estimate and compensate with less influence of the moving targets.

The frequency domain method is usually more time efficient than the image correlation one, thanks to the highly efficient FFT operation in contrast with the time-consuming image interpolation. Main steps are briefly shown as follows:

1. Transform a pair of images $s_{1}\left(t_{r},t_{a}\right)$ and $s_{2}\left(t_{r},t_{a}\right)$ into 2-D frequency domain as $S_{1}\left(f_{r},f_{a}\right)$ and $S_{2}\left(f_{r},f_{a}\right)$.
2. Remove the nominal baseline induced phase ramp as
  $$S_{2}\left(f_{r},f_{a}\right)=S_{2}\left(f_{r},f_{a}\right)\exp\left(-j2\pi f_{a}\frac{B_{xe}}{V_{e}}\right)$$
3. Estimate the interferometric phase between $S_{1}\left(f_{r},f_{a}\right)$ and $S_{2}\left(f_{r},f_{a}\right)$ over Doppler domain $f_{a}$ by averaging along range frequency $f_{r}$ to bring down noise level
  $$S_{In}\left(f_{a}\right)=\int S_{2}\left(f_{r},f_{a}\right)S_{1}{}^{*}\left(f_{r},f_{a}\right)df_{r}$$
  where ${\left(\cdot \right)}^{*}$ represents the conjugate operator.
4. Fit the residual phase of $S_{In}\left(f_{a}\right)$ with polynomials $\Delta \phi \left(f_{a}\right)=\phi_{0}+a_{1}f_{a}$ as (first order is enough for spaceborne SAR sensors with stable orbit movement)
  $$\underset{\phi_{0},a_{1}}{\min}\;{\left\|\phi_{0}+a_{1}f_{a}-∠S_{In}\left(f_{a}\right)\right\|}^{2},f_{a}\in \left[-B_{d,c}/2,B_{d,c}/2\right]$$
  where ∠⋅ denotes taking the argument, and $B_{d,c}$ is the Doppler bandwidth used for fitting.
  Calculate the effective baseline by modifying the nominal baseline with the residual baseline obtained from the first-order coefficient.
  $${\hat{B}}_{xe}=B_{xe}+\frac{a_{1}}{2\pi }V_{e}$$
  Compensate the residual phase ramp of Image 2 with the estimated phase ramp as
  $$S_{2}\prime \left(f_{r},f_{a}\right)=S_{2}\left(f_{r},f_{a}\right)\exp\left(-j\left(\phi_{0}+a_{1}f_{a}\right)\right)$$
5. Average the interferometry $S_{2}\prime \left(f_{r},f_{a}\right)S_{1}{}^{*}\left(f_{r},f_{a}\right)$ along Doppler as
  $$S_{In}\left(f_{r}\right)=\int S_{2}\prime \left(f_{r},f_{a}\right)S_{1}{}^{*}\left(f_{r},f_{a}\right)df_{a}$$
  Estimate the phase ramp induced by across-track baseline using linear regression as
  $$\underset{\phi_{0}\prime ,a_{1}\prime }{\min}\;{\left\|\phi_{0}\prime +a_{1}\prime f_{r}-∠S_{In}\left(f_{r}\right)\right\|}^{2},f_{a}\in \left[-B_{w}/2,B_{w}/2\right]$$
  Compensate the phase ramp $\phi_{0}\prime +a_{a}\prime f_{r}$ for $S_{2}\prime \left(f_{r},f_{a}\right)$ as
  $${\tilde{S}}_{2}\left(f_{r},f_{a}\right)=S_{2}\prime \left(f_{r},f_{a}\right)\exp\left(-j\left(\phi_{0}\prime +a_{a}\prime f_{r}\right)\right)$$
6. The 2-D co-registered Image 2 ${\tilde{s}}_{2}\left(t_{r},t_{a}\right)$ is obtained by 2-D IFFT of ${\tilde{S}}_{2}\left(f_{r},f_{a}\right)$.

### 3.2. Imbalance Correction

Channel imbalances mainly involve amplitude and phase responses inconsistency of the beampatterns and the following circuits. Channel imbalances can be compensated from data itself via the DB technique proposed in [25], which is updated in [3] as modified DB (MDB) to avoid degradation in low CNR scenario, which is often the case in spaceborne SAR and maritime applications. Note that the image area selected for calibration should have sufficient CNR, low number of moving targets and avoid strong moving targets like vessels. The DB/MDB algorithms are suggested to be performed in 2-D spectral domain for a limited range and Doppler bandwidths to ensure sufficient CNR [3]. The MDB algorithm is adopted in the pre-processing stage of the proposed chain. Note that before DB processing, a range power profile adaption between two channels is suggested to be performed at image or range-compressed level [3,27], which corrects the amplitude imbalances between two beampatterns in elevation.

Though DB/MDB can perform co-registration and balancing simultaneously, a prior co-registration is suggested before MDB in this paper. The co-registration in Section 3.1 is a model based approach that estimates the effective baseline in both dimensions and compensates the baseline delay via interferometric phase deramp, which is implemented for $f_{r}\in \left[-B_{w}/2,B_{w}/2\right]$ and $f_{a}\in \left[-PRF/2,PRF/2\right]$. Considering that the MDB is only performed within the calibration bandwidths, the proposed co-registration method is able to complement the MDB algorithm in exo-clutter region, where the MDB calibration is not performed. Thus, better cancellation of the exo-clutter would be expected by performing the co-registration prior to MDB.

### 3.3. Clutter Suppression

With the two SAR images co-registered and balanced, a direct DPCA subtraction is able to cancel the stationary clutter, while preserving the moving target, which can be expressed in a vector manner as

(11) $$s_{DPCA}\left(m,n\right)=w_{DPCA}{}^{H}s_{m,n}$$

where $w_{DPCA}=\frac{1}{\sqrt{2}}{\left[\begin{array}{cc} -1 & 1 \end{array}\right]}^{T}$ with the normalized factor $1/\sqrt{2}$ keeping the noise level unchanged, and the superscript H stands for conjugate transpose operation. To show the DPCA response of the azimuth ambiguities, only first order azimuth ambiguity component of $s_{m,n}$ is considered here to further derive (11) as

(12) $$\begin{array}{cc} s_{DPCA}\left(m,n\right) & =\sqrt{2}\sigma_{t}\sin\left(\frac{2\pi v_{c}B_{xe}}{\lambda V_{e}}\right)e^{j\Delta \phi_{t}/2}\\ & -\sqrt{2}\sigma_{c}^{-1}\sin\left(\frac{\pi B_{xe}f_{prf}}{V_{e}}\right)e^{j\Delta \phi_{c}^{-1}/2}\\ & +\sqrt{2}\sigma_{c}^{+1}\sin\left(\frac{\pi B_{xe}f_{prf}}{V_{e}}\right)e^{j\Delta \phi_{c}^{+1}/2}+\cdots +\tilde{n} \end{array}$$

where $\tilde{n}$ is the output noise with the same power as $n_{1}$ or $n_{2}$. It is shown in (12) that the max SNR gain for target is 3 dB in image domain. However, the target SNR gain would decrease as the across-track velocity decreases to zeros or increases to the first blind speed $v_{c}=\frac{\lambda V_{e}}{2B_{xe}}$.

As for the azimuth ambiguities, it is observed in (12) that only if the DPCA condition, $\frac{B_{xe}f_{prf}}{V_{e}}=z,z\in \mathbb{Z}$, is almost satisfied, would the azimuth ambiguity term be canceled down to zero, which would improve the SCNR at the output of DPCA filter greatly. Generally, in airborne case, the azimuth ambiguity is not a problem for GMTI because the PRF is easy to be chosen large enough to cover up to the first high sidelobe. However, in spaceborne case, the PRF could not be selected high enough to fully avoid azimuth ambiguities, which has to be in compromise with the swath width and azimuth resolution. Moreover, the DPCA condition could not be always satisfied with the same reasons. Thus, it is still necessary to handle it carefully in GMTI when the DPCA condition is not satisfied.

Other image-based method such as signal subspace projection (SSP) and joint pixel vector method (JPVM) is briefly presented here and will be compared and discussed latter. These two methods are similar by stacking the adjacent pixels of multichannel images into a vector and applying weighted filter to minimize the clutter-pulse-noise component.

The SSP data vector is constructed as $x_{SP-m,n}={\left[\begin{array}{cc} s_{1}\left(m,n\right) & s_{2-m,n} \end{array}\right]}^{T}$, where $s_{2-m,n}\in {\mathbb{C}}^{1\times N_{p}}$ is a row vector containing several pixels around (m,n) in Image 2, $N_{p}$ is the number of pixels selected. Thus, the weight vector can be derived from

(13) $$\begin{array}{l} \underset{w_{SP}}{\min}\;w_{SP}{}^{H}R_{SP-cn}w_{SP}\\ s.t.\;\;\;\;w_{SP}{}^{H}a_{SP}=1 \end{array}$$

as $w_{SP}=\mu R_{SP-cn}^{-1}a_{SP}$, where μ is a constant, $a_{SP}={\left[\begin{array}{cccc} 1 & 0 & \cdots & 0 \end{array}\right]}^{T}$ is the constraint vector, and $R_{SP-cn}=\frac{1}{L}\sum_{m,n}x_{SP-m,n}x_{SP-m,n}^{H}$ is the maximum likelihood (ML) estimated covariance matrix of clutter-plus-noise. Note that the SSP method only suppresses clutter and does not match to the across-track velocity $v_{c}$, which may introduce up to 3 dB SNR loss compared with a matched case for two spatial DOFs.

The JPVM method differs in the data vector construction by $x_{JP-m,n}={\left[\begin{array}{cc} s_{1-m,n} & s_{2-m,n} \end{array}\right]}^{T}$, where $s_{1-m,n}\in {\mathbb{C}}^{1\times N_{p}}$ is composed of the pixels in Image 1, whose indexes are the same as in $s_{2-m,n}$. The JPVM clutter suppression weight is quested by solving

(14) $$\begin{array}{l} \underset{w_{JP}}{\min}\;w_{JP}{}^{H}R_{JP-cn}w_{JP}\\ s.t.\;\;\;\;w_{JP}{}^{H}a_{JP}=1 \end{array}$$

to have $w_{JP}=\mu R_{JP-cn}^{-1}a_{JP}$, where $a_{JP}={\left[\begin{array}{ccccc} \cdots & 0 & 1 & 0 & \cdots \end{array}\right]}^{T}$ with the element corresponding to the pixel $s_{1}\left(m,n\right)$ being 1 and 0 elsewhere.

The SSP method tries to cancel the clutter in pixel $s_{1}\left(m,n\right)$ by a cluster of pixels in Image 2 $s_{2-m,n}$, which is believed to be correlated with $s_{1}\left(m,n\right)$ to improve clutter suppression. The JPVM further believes that the pixels adjacent to $s_{1}\left(m,n\right)$ in Image 1 are also correlated with $s_{1}\left(m,n\right)$, thus JPVM can be seen as cascading a secondary clutter cancellation filter to the SSP method [23]. The advantage of SSP and JPVM is that they do not call for precise co-registration or calibration to work well without much performance loss [21,22], because the co-registration and calibration are handled merged with adaptive clutter cancellation. Differently, the EDPCA and ISTAP frameworks [13,18] handle co-registration and imbalances correction via DB algorithms [25] adaptively at the pre-processing stage before subsequent adaptive clutter cancellation.

However, when the sensor array is well co-registered and calibrated by means such as the DB algorithm, SSP and JPVM may not contribute much to the SCNR improvement because more adjacent pixels do not necessarily mean “more information” for main clutter. That is to say, the correlation exploited by adaptive clutter suppression is general very weak among adjacent pixels for a focused SAR image. The weak correlation mainly comes from mainlobe oversampling and sidelobe leakage of the SAR point spread function (PSF). It also worth noting that DPCA needs the data to be well co-registered and calibrated in advance to maintain the clutter suppression performance. Note that SSP needs the covariance matrix to be estimated from a region that has sufficient CNR (or at least the same level as the area of interest) and contains low number of moving targets, so as to achieve proper clutter cancellation as well as to avoid target degradation.

### 3.4. Moving Target Detection

Generally, a 2-D CFAR detector is needed to automatically detect the moving targets. As the main clutter has been suppressed efficiently, we mainly focus on the residual ambiguous clutter. In SAR images, the responses of the moving targets and azimuth ambiguities are smeared in size and lowered in amplitude, where azimuth ambiguities are generally more severely smeared than moving targets caused by Doppler aliasing. Based on this, the size and amplitude features can be utilized and a 2-D sliding-window CFAR detector is able to distinguish them in some extent. That is, the protection window size of the detector can be determined to match with the moving targets and smooth out the azimuth ambiguities.

The size of the 2-D sliding window is determined according to the maximum smearing width of the potential moving targets. The smearing effect of moving targets is analyzed concerning the smearing widths along range and azimuth directions, respectively. The smearing width along range direction is mainly determined by range cell migration (RCM) of the moving target during synthetic aperture time $T_{a}\frac{V_{e}}{V_{e}-v_{a}}$ as

(15) $$\Gamma_{r}=v_{c}T_{a}\frac{V_{e}}{V_{e}-v_{a}}$$

in meters, where $T_{a}$ is the synthetic aperture time for stationaries. The smearing width along azimuth has been derived in condition of no azimuth ambiguity as

(16) $$\Gamma_{a}=\frac{\left|V_{e}^{2}-{\left(V_{e}-v_{a}\right)}^{2}\right|}{V_{e}^{2}}T_{a}=\left|2\frac{v_{a}}{V_{e}}-{\left(\frac{v_{a}}{V_{e}}\right)}^{2}\right|T_{a}$$

in meters.

Specifically, given $V_{e}\approx$ 7147 m/s, $T_{a}\approx$ 0.8 s. Considering a very fast ground moving target, let $v_{c}=$ 25 m/s (corresponding to a ground range velocity of about50 m/s) and $v_{a}=$ 50 m/s, which gives a 256 km/h ground speed and is high enough for most targets. This results in a $\Gamma_{r}\approx$ 20 m (or 9 range pixels) and $\Gamma_{a}\approx$ 80 m (or 30 azimuth pixels). Thus, a CFAR detector with a 2-D protection window size 12 × 30 is commonly enough for most targets.

### 3.5. Parameter Estimation

Commonly, the radial velocity of a detected target can be estimated by averaging the ATI phase of the pixels belong to the same target, which is shown as

(17) $${\hat{v}}_{c}=\frac{1}{L}\sum_{m,n\in \Omega }\Delta \phi_{m,n}\frac{\lambda V_{e}}{4\pi B_{xe}}$$

where $\Delta \phi_{m,n}$ is the interferometric phase of pixel (m,n), Ω is the collection of the pixels belong to a same target, and L is the number of pixels contained in Ω. A three-channel SAR sensor is usually used in airborne case, which provides accurate radial velocity estimation by using the ATI approach after clutter suppression. When it comes to the dual-channel spaceborne SAR sensor, the ATI approach is applied to the SAR images before clutter suppression considering limited spatial degrees-of-freedom. Note that the probability density function (*pdf*) of the ATI phase is centered at zero in absence of moving targets, while the *pdf* of the ATI phase in the presence of moving target is theoretically centered between $\Delta \phi_{t}$ toward zero, depending on the SCNR condition. Consequently, the estimation of $v_{c}$ will be biased from $\Delta \phi_{t}$ toward zero, especially for targets with low SCNR, where the clutter becomes dominant. To mitigate this bias, an adaptive matched filtering (AMF) algorithm [16,35] can be optionally applied as

(18) $${\hat{v}}_{c}=\underset{v_{c}}{\mathrm{argmax}}\;{\left\|\frac{a{\left(v_{c}\right)}^{H}R_{cn}{}^{-1}s}{a{\left(v_{c}\right)}^{H}R_{cn}{}^{-1}a\left(v_{c}\right)}\right\|}^{2}$$

where $a\left(v_{c}\right)={\left[\begin{array}{cc} 1 & e^{j\Delta \phi \left(v_{c}\right)} \end{array}\right]}^{T}$ is the steering vector, $R_{cn}=\frac{1}{L}\sum_{m,n}s_{m,n}s_{m,n}^{H}$ is the estimated covariance matrix. Equation (18) indicates that the AMF algorithm performs clutter suppression by $R_{cn}^{-1}$ and estimates the radial velocity by searching for the most matched $a\left(v_{c}\right)$ that maximizes the output SCNR of the target.

Based on the fact that along-track velocity leads to smearing and energy loss of the moving target, the along-track velocity estimation is done by searching the $v_{a}$ that maximizes the peak power of the refocused target, which is expressed as

(19) $${\hat{v}}_{a}=\underset{v_{a}}{\mathrm{argmax}\;\max}\;\left|s_{tgt}\left(m,n\right)*h\left(m,n;{\hat{v}}_{c},v_{a}\right)\right|$$

where $s_{tgt}\left(m,n\right)$ is the clutter suppressed SAR image chip that contains a moving target, ∗ denotes the convolution operation, and $h\left(m,n;{\hat{v}}_{c},v_{a}\right)$ is the matched filter that focuses the smeared moving target, with ${\hat{v}}_{c}$ obtained from Equation (18) and along-track velocity $v_{a}$ assumed unknown for searching.

## 4. Results and Discussions

The data are measured by Gaofen-3 SAR sensor with DRC GMTI experimental mode, and the system parameters are listed in Table 1. In this experiment, a scene that contains a highway near the Chinese Yangtze River is selected to assess the GMTI performance of the Gaofen-3 dual-channel SAR sensor. The SAR image of the selected scene is shown in Figure 1, where the range direction (look direction) is descending and the azimuth direction towards right. Note that the tilted highway is on the left part of the image, while the river with several vessels is on the right part. It can be observed that the reflectivity of these vessels is very strong, which will cause significant azimuth ambiguities with shifted azimuth positions to the ground part. Although the strong azimuth ambiguities are almost invisible in the imagery due to controlled AASR, they may still appear in the clutter suppressed map, which will be shown later.

### 4.1. Overall SAR-GMTI Results for Gaofen-3

In this subsection, we mainly focus on the highway region as shown in Figure 1. The image-based SAR-GMTI process is applied to this region according to the workflow as shown in Figure 3. Overall results are presented in the following to preliminarily demonstrate the GMTI capability of the Gaofen-3 dual-channel SAR sensor.

**Figure 4:**
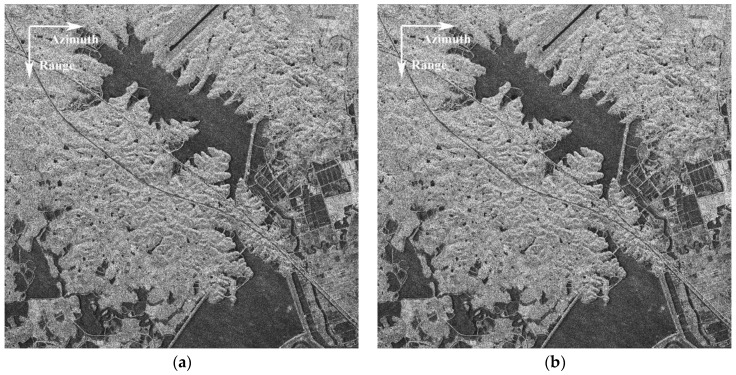
SAR imagery of the highway region: (**a**) fore-channel image; and (**b**) aft-channel image.

In Figure 4, the two co-registered images are presented, where Figure 4a is the SAR image of the fore-channel and Figure 4b is the image obtained from the aft-channel. It can be seen that the two images show very good consistency in amplitude, which indicates excellent channel calibration of the Gaofen-3 SAR sensor, which will be further analyzed latter. By observing the scene in Figure 4, it is seen that the ground moving targets, e.g., vehicles, are buried in the strong land clutter and are hardly identified with eyes, which is also due to that ground moving targets like vehicles are usually very small in size and weak in reflectivity. Note that the road is tilted in the image, which indicates that the moving targets on the road would have both moderate along-track and across-track velocities.

**Figure 5:**
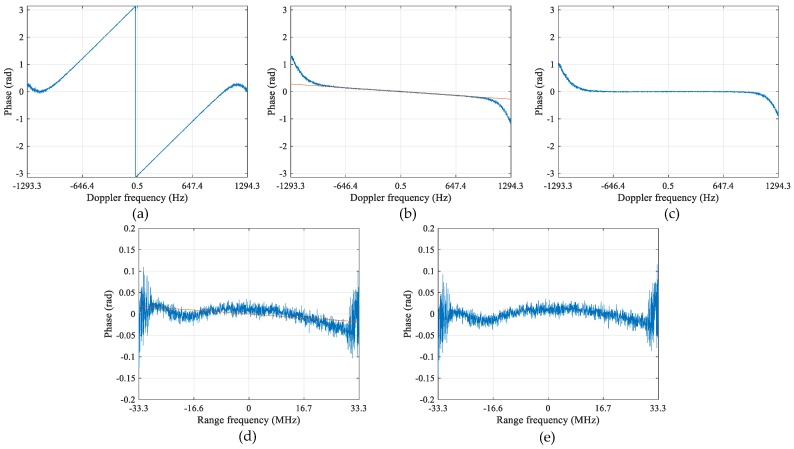
Interferometric phase in Doppler domain (**a**–**c**); and interferometric phase in range frequency domain after along-track co-registration (**d**,**e**): (**a**) before co-registration; (**b**) after coarse along-track baseline co-registration; (**c**) after residual along-track baseline co-registration; (**d**) phase in range frequency domain after along-track co-registration; and (**e**) after across-track co-registration.

It is a key technique to obtain the well co-registered image pair, as shown in Figure 4, which is done by compensating the interferometric phase slope in range frequency and Doppler domains (2-D spectral domain) here, as presented in Section 3. Figure 5 shows the intermediate results of the interferometric phase during co-registration procedure, where Figure 5a is the interferometric phase in Doppler domain before co-registration, which is obtained by averaging the interferometric phase along range frequency direction in the 2-D spectral domain; Figure 5b is the interferometric phase after coarse co-registration according to system geometry; and Figure 5c is obtained by further compensating the phase using the estimated residual baseline obtained from Figure 5b. It can be seen in Figure 5a that the unwrapped interferometric phase is actually proportional to Doppler frequency with the wrapping point around zero Hz. In Figure 5b, it is observed that the slope ratio alters from negative of Figure 5a to positive after coarse co-registration, which implies that the real baseline is slightly shorter than the nominal one. The practical factors leading to it have been mentioned in Section 3. In Figure 5b, the red line represents the fitted phase line with the middle Doppler band as samples. The zero-centered and flat phase curve in Figure 5c indicates good co-registration of the along-track baseline. It is also observed in Figure 5c that an asymmetric phase deviation tendency appears at the left and right edges in the Doppler domain, which is the impact of a higher ambiguous ratio in these regions.

Figure 5d,e is the interferometric phases in range frequency domain, which are obtained by averaging along Doppler direction for each range frequency bin. Figure 5d is the phase after along-track baseline co-registration, where a slow varying phase trend in range frequency domain is observed. The red line in Figure 5d presents the linearly fitted phase ramp along range. Figure 5e shows the phase in range frequency domain after deramp processing using the phase ramp estimated in Figure 5d. Note that there are still some phase imbalances in Figure 5e, which will be compensated in the following imbalance correction stage.

**Figure 6:**
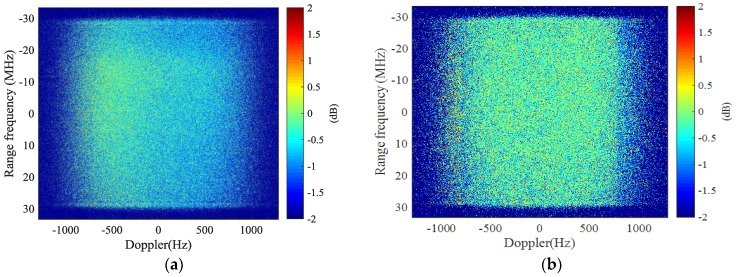
Amplitude imbalances in the 2-D spectral domain before and after imbalance correction (calibration bandwidths: 60 MHz in range and 1.58 kHz in Doppler): (**a**) before imbalance correction; and (**b**) after imbalance correction.

The channel imbalances before and after imbalance correction are assessed using a useful metric proposed in [36]. Figure 6 shows the amplitude imbalances in 2-D spectral domain before and after imbalance correction using MDB algorithm, where Figure 6a is the amplitude imbalances before correction and Figure 6b is the amplitude imbalances after MDB. Some non-consistency is observed in Figure 6a via the non-uniform distribution of the amplitude in 2-D spectral domain. The asymmetric distribution may be caused by slightly different squint angle between two antennas. Comparing Figure 6a and Figure 6b, it is observed that the amplitude imbalances of Figure 6b have become more uniformly distributed than that of Figure 6a, which indicates that better amplitude consistency is obtained after correction. Quantitatively, the average imbalances before correction is −0.6356 dB for Figure 6a, which is reduced to −0.3441 dB after correction for Figure 6b. According to the definition in [36], amplitude imbalances should be 0 dB for ideal case. These figures are fairly satisfactory for a spaceborne dual-channel SAR sensor. In addition, the MDB algorithm is effectively implemented in this case. Note that the amplitude imbalances are averaged for range bandwidth of 60 MHz and Doppler bandwidth of 1580 Hz, which are also the calibration bandwidths. It is also noticed in Figure 6b that there are some slight degradation outside the calibration bandwidths (above 790 Hz), which is caused by a higher ambiguity ratio and some uncorrected imbalances in this region.

**Figure 7:**
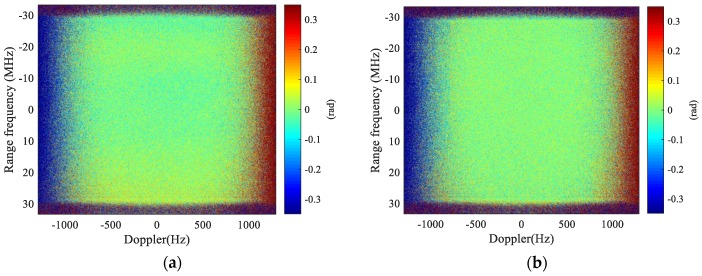
Phase imbalances in the 2-D spectral domain before and after imbalance correction (calibration bandwidths: 60 MHz in range and 1.58 kHz in Doppler): (**a**) before imbalance correction; and (**b**) after imbalance correction.

The phase imbalances are assessed similarly as the amplitude. Figure 7 presents the phase imbalances before and after correction, where Figure 7a gives the phase imbalances before correction and Figure 7b is the phase imbalances after correction. The phase imbalances in 2-D spectral domain can be observed to be well corrected as a more uniform phase distribution is obtained in Figure 7b. The average phase imbalances before correction is −0.3441 degree within the support area, and the imbalances are brought down to −0.0076 degree. This indicates excellent phase consistency between channels after correction.

**Figure 8:**
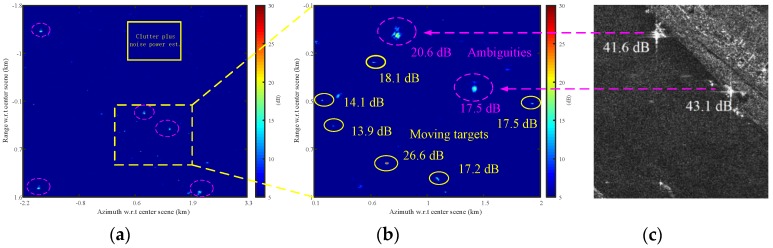
Signal-to-clutter-plus-noise ratio (SCNR) image using signal subspace projection (SSP) with a 2-by-2 magnitude multilook (The clutter-plus-noise power is estimated by averaging the pixels within the solid rectangle box in (**a**)): (**a**) full scene; (**b**) magnified part of (**a**); and (**c**) objects that cause ambiguities in (**b**) (the image chip is taken from the lower right part of Figure 1).

Figure 8 presents the clutter cancellation results using SSP method with a 2-by-2 magnitude multilook. Figure 8a is the full scene and Figure 8b is the magnified part of Figure 8a as indicated by the dashed box. Note that Figure 8a,b is displayed in terms of SCNR as indicated by sidebar, where the region delimited by the solid box in Figure 8a is used to estimate the clutter-plus-noise power as well as to estimate the interference covariance matrix for SSP. In Figure 8b, several moving targets are manually marked with yellow solid circle and their SCNR included.

In addition, two ambiguities are marked in Figure 8b with dashed circle, which come from the strong objects as shown in Figure 8c, which is found on the lower right part of Figure 1. The strong objects are about 40 dB above the river region, and their first ambiguities appear in the clutter canceled map with around 20 dB above the background as shown in Figure 8b. Thus, the ambiguities are so strong to greatly impact the detection of the moving targets.

**Figure 9:**
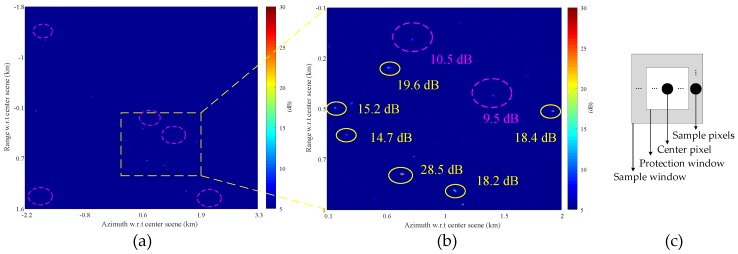
Clutter cancellation result in term of output SCNR (the clutter-plus-noise power is estimated for each pixel from adjacent pixels, which are selected by the sliding window of the constant false alarm rate (CFAR) detector): (**a**) full scene; (**b**) magnified part of (**a**); and (**c**) clutter-plus-noise power estimation.

Figure 9 displays the same clutter cancellation result as shown in Figure 8. Figure 9 differs from Figure 8 in that the clutter-plus-noise power is estimated for each pixel by averaging the power of pixels surrounding it as shown in Figure 9c, where a protection window size 12-by-20 and a sample window size 22-by-30 are employed here. Thus, Figure 9 is presented by estimating the SCNR for each pixel with the clutter-plus-noise power estimated from surrounding sample pixels as indicated by Figure 9c. It is observed from Figure 9b that the re-estimated SCNR of the ambiguities are averagely about 9 dB lower than Figure 8b, while the SCNR of the moving targets are around 1 dB higher. The degradation of the ambiguities in Figure 9b can be regarded as the result of an efficient estimation for the clutter texture (slow variant clutter-plus-noise power in Figure 8) with the sliding window shown in Figure 9c.

**Figure 10:**
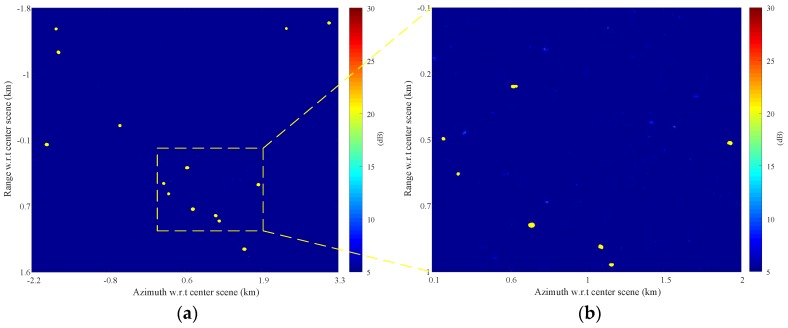
CFAR Detection result with the detected pixels marked by yellow dots: (**a**) full scene; and (**b**) magnified part of (**a**).

Figure 10 is the detection result obtained by a CA-CFAR detector with the same protection and sample windows as shown in Figure 9c under the assumption of Gaussian distribution. Clutter-plus-noise power is estimated by averaging the power of the sample pixels, and then a threshold assuming a probability of false alarm $P_{fa}$ of $10^{-6}$ is set up for detection. Detected pixels are marked as yellow dots in Figure 10a,b. Figure 10 is also displayed in terms of SCNR with the clutter-plus-noise power estimated from sample pixels delimited by protection and sample windows, which is the same as in Figure 9. Consequently, the lowered SCNR estimation of the azimuth ambiguities prevents these pixels from being detected.

**Figure 11:**
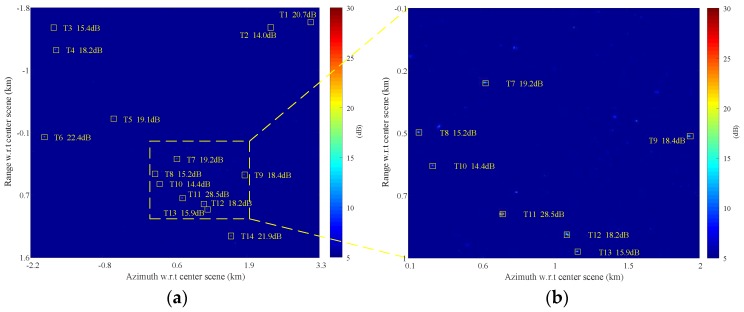
Clustering result of Figure 10: (**a**) full scene; and (**b**) magnified part of (**a**).

Figure 11 presents the clustering result, where the detected pixels (as shown in Figure 10) that belong to the same target are clustered together. Each clustered target is marked by a small rectangle with its SCNR denoted aside in Figure 11a,b.

**Figure 12:**
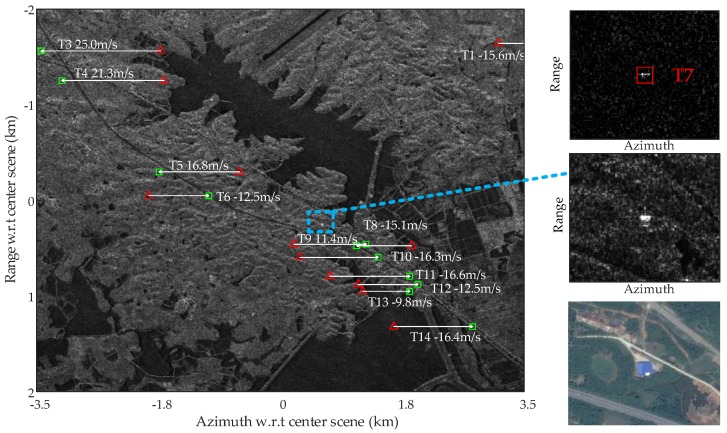
Relocation map of the moving targets with ground radial velocities estimated by adaptive matched filter (AMF) (upper chip is the SSP output of Target 7, middle chip is the magnified SAR image containing Target 7, and lower chip is the optical image corresponding to the middle chip).

In Figure 12, the detected moving targets are relocated to their real positions via estimation of the radial velocity using the AMF method, where the red triangles mark the position of the moving targets detected, and the green squares represents the relocated real position of the moving targets. The radial velocities of the moving targets are estimated using the AMF approach according to Equation (18), and the moving targets deviation in azimuth can be calculated using the estimated radial velocity as

(20) $$\Delta X=\frac{{\hat{v}}_{c}}{V_{e}}R_{s}$$

where $R_{s}$ is the slant range of the target, ${\hat{v}}_{c}$ is positive when approaching radar, and ΔX is positive when the target is shifted right in image. Note that the unit of ΔX is meter, alternatively ΔX can be written in terms of pixel as

(21) $$\Delta X=\frac{{\hat{v}}_{c}R_{s}f_{prf}}{V_{e}^{2}}$$

It is seen in Figure 12 that most targets are relocated correctly to the highway, while a few targets are relocated with some error off the road. This verifies that the radial velocity estimation of Gaofen-3 satellite is potentially excellent. However, it is still interesting to analysis the existing limitations in Figure 12. Note that the chips beside Figure 12 magnify the Target 7 in clutter canceled image, original SAR image, and optical image. The upper chip of Figure 12 is the magnified clutter cancellation output for Target 7; the middle chip is the magnified SAR image for Target 7; and the lower chip is the optical image for Target 7 obtained from Google Earth. It is possible that Target 7 in the upper chip is the residual clutter of the strong stationary object. It is also possible that the Target 7 shown in the upper chip is a detected moving target but fails to be relocated in Figure 12. In this case, the azimuth shifted Target 7 may happen to be overlapped with and masked by a strong stationary object in the SAR image, so that the interferometric phase of Target 7 is wrongly estimated and biased to the dominant stationary component. With the estimated radial velocity too close to zero, we screen Target 7 out of the relocation result and deem it as a false alarm. Actually, the relocation accuracy relies on SCNR of the target before clutter suppression to a large extend. Thus, even if the clutter can be totally canceled, there are chances that a weak target can be detected after clutter suppression but fails to be relocated using interferometry.

Table 2 details the quantitative analysis of all detected targets, where estimated across-track ground velocity, estimated ground-truth of the velocity and estimation error are concerned. The results shown in Table 2 are obtained in condition that the SSP algorithm is used for clutter cancellation and AMF for across-track estimation. It worth noting that the estimated ground-truth is obtained by assuming that the targets have been precisely relocated to the road, so that the azimuth shift can be used to infer the real across-track velocity; however, this estimated ground-truth is not precisely the real one, because the azimuthal displacement is not only related to across-track velocity but also affected by factors such as across-track acceleration and along-track velocity [34]. The across-track ground velocity is expressed as ${\hat{v}}_{c}/\sin\left(\theta_{i}\right)$, where $\theta_{i}$ is the incident angle. In addition, estimated input and output SCNRs of each target are also listed in Table 2, and the improvement factor (IF) is given by $IF=SCNR_{out}/SCNR_{in}$.

**Figure 13:**
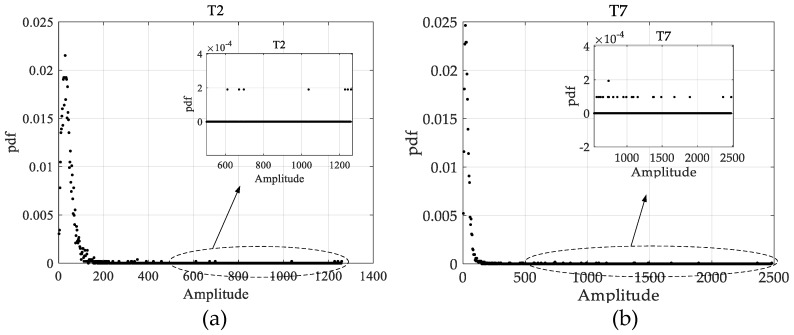
Statistical distribution of clutter around Targets 2 and 7 in the SAR image before clutter cancellation: (**a**) clutter distribution around Target 2; and (**b**) clutter distribution around Target 7.

In Table 2, Targets 2 and 7 are seen as false alarm and marked asterisk. The estimated input SCNRs of Targets 2 and 7 are fairly high for 26.5 and 31.78 dB, the case of Target 2 is similar to Target 7 as shown in Figure 12. To explain the high input SCNRs of Targets 2 and 7 more clearly, we present the statistical distributions of the clutter around Targets 2 and 7 in Figure 13a,b, respectively, where a clutter patch of 50-by-50 pixels around the interested target is used to obtain the clutter distribution. It is observed in Figure 13a,b that the distributions around Targets 2 and 7 both have long tails, which is in accordance with the high input SCNRs of Targets 2 and 7 as shown in Table 2. The input SCNRs of Targets 3 and 8 are relatively low, that is 3.45 dB and 6.67 dB, which result in their increased estimation errors of 2.8 and −3.28 m/s. It is observed that targets with input SCNR above 10 dB possess a high probability to obtain radial ground velocity estimation error below 1 m/s, such as Targets 1, 6, 9–12 and 14.

**Figure 14:**
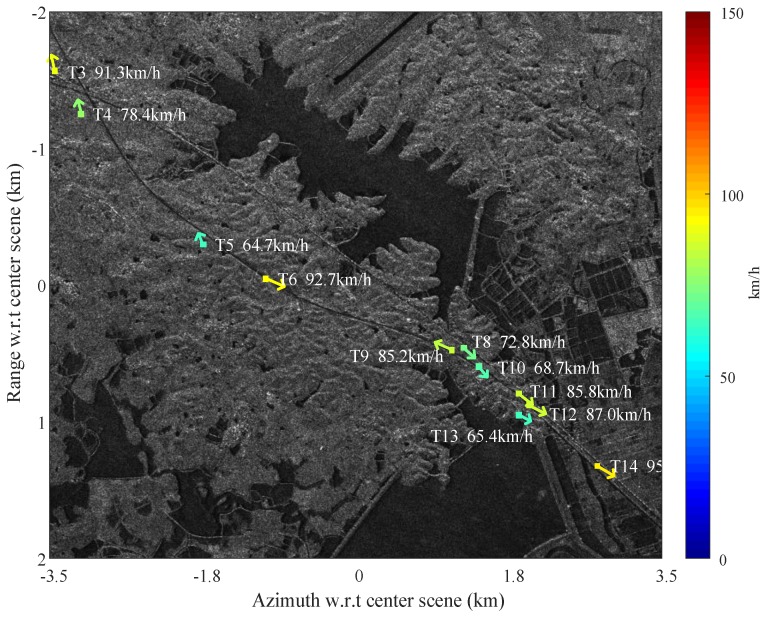
Vector velocity map of the moving targets (colors code the modulus ground velocity of the moving targets in km/h).

Figure 14 shows the vector velocity map of the moving targets. This is achieved by additional estimation of the along-track velocity of the target, where the clutter canceled moving target chip is extract from clutter canceled image, and a parameter estimation is done to search for the along-track velocity that maximize the target energy. This method is based on the fact that along-track velocity of the moving target results in target azimuth smearing in the SAR image, thus energy loss. The color bar of Figure 14 denotes the ground velocity of the moving targets, with unit being km/h. It can be seen that all velocity vectors of the moving targets are along the road trend, which indicates effective estimation of both radial and along-track velocities of the moving targets. The along-track velocity estimation may be more stable considering that it can be estimated from the clutter canceled data where the SCNR is improved greatly.

**Figure 15:**
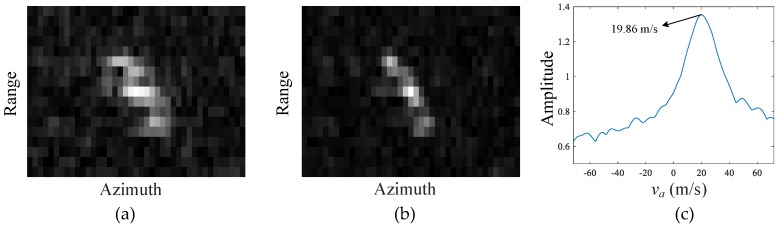
Along-track velocity estimation and refocusing of Target 12: (**a**) unfocused moving target; (**b**) refocused moving target; and (**c**) along-track velocity search curve.

To demonstrate the along-track velocity estimation of the moving target, Target 12 in Figure 15 is taken as an example. Figure 15a is the smearing moving target after clutter suppression, Figure 15b is the refocused moving target with the searched along-track velocity and afore-obtained radial velocity, and Figure 15c is the searching curve of the along-track velocity, where the estimated along-track velocity is obtained at the maximum amplitude point. It can be seen in Figure 15b that the moving target is well focused with significant image feature, which indicates the efficient estimation of the vector velocity. It is also interesting to see that this target may be a long truck with its body axis along the tilted road as shown in Figure 14.

### 4.2. Discussion on Clutter Suppression

**Figure 16:**
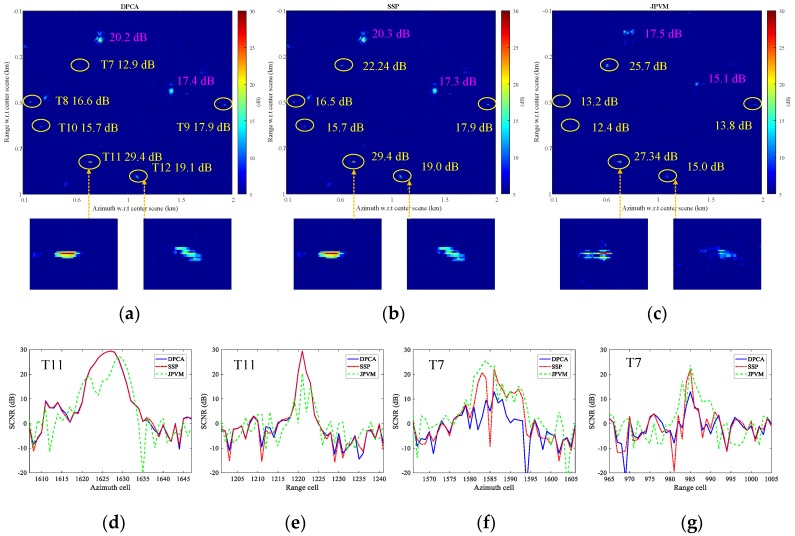
Clutter suppression comparison results (without multilook): (**a**) displaced phase center antenna (DPCA); (**b**) SSP; (**c**) joint pixel vector method (JPVM); (**d**) azimuth cut of Target 11; (**e**) range cut of Target 11; (**f**) azimuth cut of Target 7; and (**g**) range cut of Target 7.

To compare the clutter suppression for DPCA, SSP and JPVM, a small region is taken for comparison. The clutter suppression is evaluated in terms of output SCNR for the targets. The three methods are all implemented to well co-registered and balanced images, and no multilook is performed. Figure 16a–c presents the clutter cancellation results of DPCA, SSP, and JPVM, respectively, while the magnified image chips of Targets 11 and 12 are also shown below the SCNR maps. It can be observed from Figure 16a–c that DPCA and SSP almost give the same output SCNR for Targets 8–12, while DPCA and SSP are averagely better than JPVM for about 3 dB for Targets 8–12 in terms of SCNR. For Target 7, SSP nearly provide 10 dB better SCNR gain than DPCA.

As for ambiguities, it is seen in Figure 16c that JPVM suppresses the ambiguous clutter better than DPCA and SSP. Specifically, the ambiguous clutter patches shown in Figure 16c are decreased in size and average power compared to those of Figure 16a,b; nonetheless, the peak SCNR of the ambiguities in Figure 16c is decreased by about 3 dB compared to Figure 16a,b.

**Figure 17:**
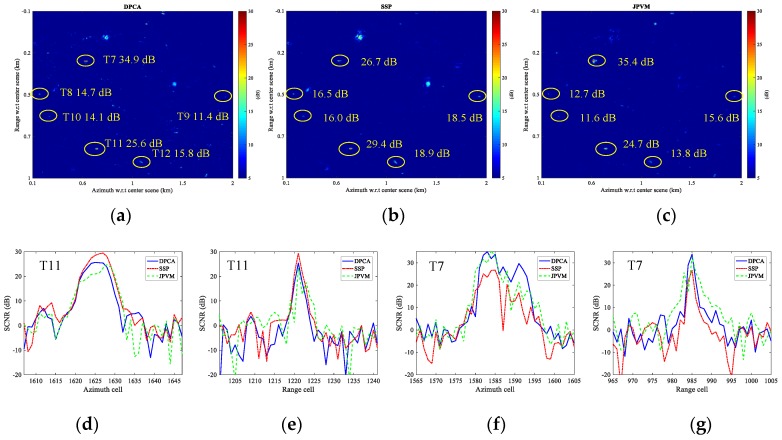
Clutter suppression comparison results with co-registration error of 0.2 pixel (without channel balancing and multilook): (**a**) DPCA; (**b**) SSP; (**c**) JPVM; (**d**) azimuth cut of Target 11; (**e**) range cut of Target 11; (**f**) azimuth cut of Target 7; and (**g**) range cut of Target 7.

As for moving targets, it is observed in Figure 16a,b that the target responses of Targets 11 and 12 are well preserved after clutter cancellation using DPCA and SSP, while the target response obtained by JPVM in Figure 16c shows a clear distortion, which would not only impair the SCNR of the moving target but also impedes imaging the moving target clearly with the clutter canceled data. The response distortion and SCNR degradation of the target are also observed in [23], which derived the relationship between SSP and JPVM. The processing result in [23] shows an output SCNR loss around 10 dB for JPVM with respect to SSP concerning a moving target with 30 dB input SCNR, where *N_p_* equals 7-by-7 for JPVM and the data are processed without co-registration and balancing. A comparable result is shown in Figure 17c, where a SCNR loss of 5 dB is observed for Target 11 with an input SCNR of 18.8 dB and *N_p_* equals 3-by-3. Note that *N_p_* of 7-by-7 almost gives the same result as Figure 17c and is not given here.

Range and azimuth cuts of Figure 16a–c are also given here. Figure 16d,e gives the azimuth and range cuts of Target 11, while Figure 16f,g gives the cuts of Target 7. In Figure 16d,e, JPVM is observed to distort the point spread function of the moving target (biased peak position and notches in middle), and the output SCNR of Target 11 is also degraded for JPVM with respect to DPCA and SSP. Targets 8 through 12 basically behave the same as Target 11, so that they are not presented here due to space limitations. Note that it is an exception for Target 7 as shown in Figure 16f,g, and it is not fully sure whether the higher SCNR obtained by JPVM results from true SCNR improvement or wrongly estimated SCNR corrupted by residual clutter (the discrete clutter masking Target 7 is about 30 dB higher than adjacent land clutter in original image).

It is also interesting to compare the three methods without precise co-registration and channel balancing. The original images are only pre-processed with an integer azimuth shift of 1 pixel to achieve coarse co-registration (result in co-registration error of about 0.2 pixel) before clutter suppression. Figure 17a–g give the comparison results holding the same meaning as Figure 16, where co-registration and imbalance correction are implemented before clutter suppression. It can be observed in Figure 17a that DPCA suffers from SCNR degradation ranging from 1.6 to 6.5 dB compared with Figure 16a, while SSP almost gives unchanged output SCNR in presence of co-registration error and channel imbalance compared with Figure 16b. JPVM has slight degradation of 0.5, 0.8, 2.6, and 1.2 dB for Targets 8, 10, 11, and 12, respectively, compared with Figure 16c. Target 9 is improved by 1.8 dB for JPVM and 0.6 dB for SSP than Figure 16. This comparison verifies that SSP and JPVM are robust to co-registration error and imbalances to some extent, and that SSP is better performed than JPVM in terms of SCNR. Note that Target 7 is covered by a strong stationary object in original image, so that the outputs of DPCA and JPVM on this point are likely to be raised by the residual clutter rather than moving target.

From the presented results in Figure 16 and Figure 17, JPVM does not seem to perform comparable with SSP in terms of output SCNR and IF, so that the JPVM is not suggested to be included into our processing chain as shown in Figure 3.

### 4.3. Discussion on Across-Track Velocity Estimation

**Figure 18:**
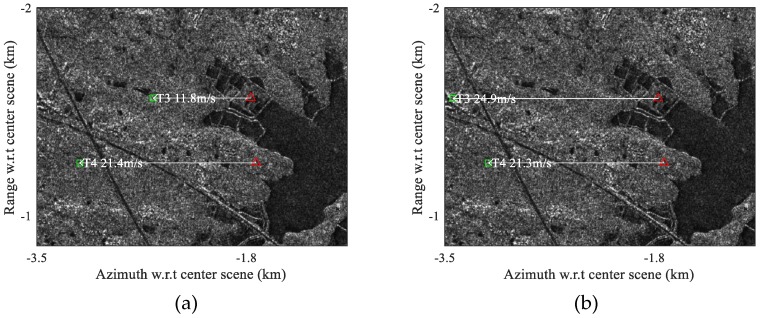
Comparison of across-track velocity estimation for along-track interferometry (ATI) and AMF: (**a**) original ATI phase is used to estimate across-track velocity; and (**b**) AMF is used to estimate target’s ATI phase and across-track velocity.

It is mentioned in Section 3.5 that a direct estimation of the across-track velocity using ATI phase as in Equation (13) is a biased estimation, and that AMF estimation (Equation (14)) is less biased, especially for low SCNR condition. Here, we have taken Target 2 for comparison, whose input SCNR is relatively low, 3.45 dB, as listed in Table 2. Figure 18a presents the across-track velocity estimation using original ATI phase, Figure 18b estimates the across-track velocity and the ATI phase of Target 2 using AMF. It is observed in Figure 18a that in low SCNR condition, the across-track velocity estimation deviates from real value towards zero seriously, which is induced by non-negligible clutter component whose ATI phase is zero. In Figure 18b, less biased from the road is seen, which indicates that AMF estimator is less biased compared with a direct interferometry for low SCNR case.

### 4.4. Discussion on CFAR Detection

It is observed in Figure 16 that the azimuth strong ambiguity appears as a big patch in shape with size about 20-by-30 in range and azimuth, respectively. The amplitude of the ambiguity patch is relatively flat. Thus, a CFAR detector with the designed window size is likely to regard the azimuth ambiguities as fluctuation background and smooth them away in the sliding process.

**Figure 19:**
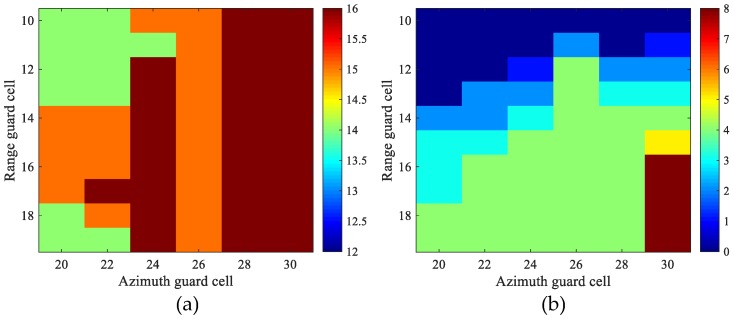
Number of detected targets/ambiguities versus range/azimuth guard cell size: (**a**) number of detected targets; and (**b**) number of detected ambiguities.

To assess the performance of the proposed detection strategy, we change the size of the guard window both in range and azimuth, while the outer window that delimits the clutter sample is always 5 pixels larger than the protection window in each dimension. According to the detection results, we manually discriminate the moving targets from ambiguities. Thus, the numbers of detected targets/ambiguities are obtained. Note that we count the number of detections after clustering rather than the number of detected pixels. Figure 19a is the number of detected targets versus range guard cell and azimuth guard cell. Figure 19b is the number of detected ambiguities (number of patch). The data in Figure 19a,b is also listed in Table 3. It can be observed from the table that the number of detected targets basically remains the same as the size of the protection window changes, while the number of detected ambiguities increases significantly as the window size increases. From the boundary in Figure 19b where the ambiguity detections start to appear, we find it safe for a 10 × 30 protection window to cover a very high-speed ground moving target, which is a little smaller than the 12 × 30 smearing width given in Section 3.4. To be more conservative, a 10 × 20 protection window size is also favorable, which may in exchange misses some moving targets with very high along-track velocity.

## 5. Conclusions

In this paper, we presented the first GMTI experimental results for the Chinese Gaofen-3 satellite operating in dual-channel GMTI experimental mode, which is designed based on FSI mode. The channel consistency of the dual-channel SAR sensor is assessed using land clutter data, which shows around −0.6 dB amplitude imbalance and 0.14 degree phase imbalance. The imbalances are further reduced to −0.3 dB and −0.007 degree using MDB algorithm. Image domain clutter suppression results indicate that azimuth ambiguities have to be considered when the DPCA condition is not satisfied, especially when strong objects are presented near the scene. Clutter cancellation performances are compared for DPCA, SSP and JPVM. Comparison results shows that DPCA and SSP perform closely in homogeneous scene when data are well co-registered and balanced, while JPVM is better at suppressing azimuth ambiguities but would cause target distortion and an SCNR loss about 3 dB or more compared to DPCA and SSP in this scene, so that it is not included as an option in the proposed processing chain. As for detection, size information and slow spatial variation feature of the ambiguities is utilized, so that a 2-D sliding window with size adapted to target smearing width helps to smooth out strong ambiguities and preserve small targets. Typically, 10 dB suppression is obtained for ambiguities in the presented example. Parameter estimation is done for both across- and along-track velocities, and relocation result and vector velocity map of the moving targets are presented to verify the GMTI capability of the Gaofen-3 dual-channel SAR sensor. It is observed that the moving targets with input SCNR above 10 dB could achieve an across-track velocity accuracy better than 1 m/s with a big chance for Gaofen-3. It is also notable that this processing chain is based on SAR image, i.e., images focused with stationary world matched filter, so as to be suboptimal for SNR loss of the moving targets which are not fully compressed, compared with the optimal but more computationally loaded ones [13,18]. In future investigations of SAR-GMTI for Gaofen-3 mission, research will involve GMTI in different imaging modes, as well as polarimetric information utilization for moving target detection.

## Figures and Tables

**Table 1:** Acquisition parameters.

Symbol	Parameter	Value
*λ*	Wavelength	0.056 m
*V_s_*	Satellite Velocity	7569.5 m/s
*θ_L_*	Look Angle	30.77°
*B_w_*	Bandwidth	60 MHz
*f_s_*	Sampling Rate	66.66 MHz
*B_x_*	Along-track Baseline	3.75 m
*f_prf_*	PRF	2588.57 Hz
*B_d_*	Doppler Bandwidth	1482.3 Hz

**Table 2:** Across-track velocity estimation result for detected targets (the clutter cancellation is performed using SSP and the across-track velocity is estimated using AMF; asterisk denotes false detection).

Targets	Est. Across-Track Ground Velocity (m/s)	Est. Ground-Truth (m/s)	Err (m/s)	SCNR_in_ (dB)	SCNR_out_ (dB)	IF (dB)
T1	−15.59	−16.14	0.54	12.02	20.74	8.72
T2*	−0.13	-	-	26.50	13.96	−12.54
T3	24.95	22.15	2.8	3.45	15.36	11.91
T4	21.30	18.77	2.53	10.94	18.25	7.31
T5	16.81	14.37	2.44	7.84	19.06	11.22
T6	−12.51	−12.54	0.02	19.85	22.45	2.6
T7*	0.18	-	-	31.78	19.21	−12.57
T8	−15.05	−11.77	−3.28	6.67	15.24	8.57
T9	11.43	11.93	−0.50	10.21	18.16	7.85
T10	−16.32	−15.88	0.44	7.77	14.36	6.59
T11	−16.58	−16.10	−0.49	18.76	28.49	9.73
T12	−12.45	−12.56	−0.11	11.76	18.23	6.478
T13	−9.78	−13.24	3.45	10.05	15.87	5.82
T14	−16.40	−15.72	−0.68	18.06	21.85	3.79

**Table 3:** Numbers of ambiguity/target detections versus range/azimuth guard cell size.

Ambiguities/Targets		Azimuth Guard Cells					
		20	22	24	26	28	30
**Range Guard Cells**	**10**	0/14	0/14	0/15	0/15	0/16	0/16
	**11**	0/14	0/14	0/14	2/15	0/16	1/16
	**12**	0/14	0/14	1/16	4/15	2/16	2/16
	**13**	0/14	2/14	2/16	4/15	3/16	3/16
	**14**	2/15	2/15	3/16	4/15	4/16	4/16
	**15**	3/15	3/15	4/16	4/15	4/16	5/16
	**16**	3/15	4/15	4/16	4/15	4/16	8/16
	**17**	3/15	4/16	4/16	4/15	4/16	8/16
	**18**	4/14	4/15	4/16	4/15	4/16	8/16
	**19**	4/14	4/14	4/16	4/15	4/16	8/16
